# Analysis on the Bus Arrival Time Prediction Model for Human-Centric Services Using Data Mining Techniques

**DOI:** 10.1155/2022/7094654

**Published:** 2022-09-26

**Authors:** N. Shanthi, Sathishkumar V E, K. Upendra Babu, P. Karthikeyan, Sukumar Rajendran, Shaikh Muhammad Allayear

**Affiliations:** ^1^Department of Computer Science and Engineering, Kongu Engineering College, Perundurai, Erode, India; ^2^Department of Industrial Engineering, Hanyang University, 222 Wangsimni-ro, Seongdong-gu, Seoul 04763, Republic of Korea; ^3^Department of Computer Science and Engineering, Bharat Institute of Higher Education and Research, Chennai, Tamil Nadu, India; ^4^School of Information Technology and Engineering, Vellore Institute of Technology, Vellore, Tamil Nadu, India; ^5^Department of Multimedia and Creative Technology, Daffodil International University, Daffodil Smart, Khagan, Ashulia, Dhaka 1207, Bangladesh

## Abstract

The human-computer interaction has become inevitable in digital world. HCI helps humans to incorporate technology to resolve even their day-to-day problems. The main objective of the paper is to utilize HCI in Intelligent Transportation Systems. In India, the most common and convenient mode of transportation is the buses. Every state government provides the bus transportation facility to all routes at an affordable cost. The main difficulty faced by the passengers (humans) is lack of information about bus numbers available for the particular route and Estimated Time of Arrival (ETA) of the buses. There may be different reasons for the bus delay. These include heavy traffic, breakdowns, and bad weather conditions. The passengers waiting in the bus stops are neither aware of the delay nor the bus arrival time. These issues can be resolved by providing an HCI-based web/mobile application for the passengers to track their bus locations in real time. They can also check the Estimated Time of Arrival (ETA) of a particular bus, calculated using machine learning techniques by considering the impacts of environmental dynamics, and other factors like traffic density and weather conditions and track their bus locations in real time. This can be achieved by developing a real-time bus management system for the benefit of passengers, bus drivers, and bus managers. This system can effectively address the problems related to bus timing transparency and arrival time forecasting. The buses are equipped with real-time vehicle tracking module containing Raspberry Pi, GPS, and GSM. The traffic density in the current location of the bus and weather data are some of the factors used for the ETA prediction using the Support Vector Regression algorithm. The model showed RMSE of 27 seconds when tested. The model is performing well when compared with other models.

## 1. Introduction

Human-computer interaction is generally defined as “the science concerned with designing effective interaction between users and computers and the construction of interfaces that support this interaction.” The digital transformation has made computers inseparable from human life. Humans are dependent on digital gadgets like mobile phones, tablets, laptops, and so on to complete their day-to-day tasks. The increase in demand for human-computer interaction has opened a wide scope of research in recent years. In terms of computer programming, many front end frameworks have been introduced to develop user-friendly mobile- or web-based applications. These applications help humans to simplify their regular tasks like paying bills, buying groceries or medicines, booking cabs, and so on. Another advantage is that even users with no technical background can also use these applications for their needs [1].

In India, the most used and convenient mode of transportation is the buses. Though this is the most used transportation, its travelers do not know the information like what the arrival time and the exact routes and available bus in the route. Passengers are supposed to wait for an indefinite time not knowing about the bus they are waiting for and information regarding the bus. Because of lack of real-time information regarding bus location, route, and traffic information, it is hard for managing the bus services for drivers, travelers, bus managers, and policy makers. To address the problems faced by the bus user's bus managers and policy makers, an Intelligent Transportation framework is proposed [2]. Initially, a data collection module that collects information about routes, timestamp, real-time bus location, traffic information, and weather information is created. Once the data collection module is developed, by using Artificial Intelligence techniques, various useful patterns such as trip duration, arrival time, trip duration, transit pattern, bus scheduling, traffic management, and so on can be identified. All the possible information regarding the transportation is made transparent for the benefit of bus users, bus managers, bus drivers, and policy makers to keep informed about a trip. By developing such a transparent system, it is possible to reduce various problems, such as traffic congestion, waiting time, transit problem, scheduling problems, and so on. The main objective of this project is to develop a real-time bus management system using Artificial Intelligence for the benefit of bus users, bus drivers and bus managers which can effectively address the problems related to bus information system transparency [3].

The ITS application specifically aimed to develop for the Indian transportation system, as an easily accessible tool with various options for passengers, bus managers, and policy makers. Despite the availability of ITS applications, there are very few trial versions of transportation applications for traffic monitoring available and no specific tool combining all the tasks of ITS. An initiation is needed to develop a project combining all aspects of ITS in India [4]. If the idea of ITS is implemented, major problem of traffic congestion can be reduced in a considerable manner. Regardless in the developed countries, India needs ITS infrastructure development to tackle the upcoming technological advancements in ITS. India is land of various languages with several cultural practices. Proper information regarding the public transportation in all the states should be developed so that people traveling to a different city from their native city can use that information for their mobility. Because of the lack of public transportation information, travelers are facing lots of difficulties while traveling from one place to another. Peoples can be easily fooled by the false information conveyed to them by unknown authorities. So, an authentic public transportation information delivery system should be developed. This information will be more useful for passengers traveling in any part of the nation irrespective of their language barrier. As a first step, a data collection framework that collects all possible information from public vehicles, road and traffic scenario is required to develop an ITS system, which can provide transparent data about vehicle patterns to the bus managers, passengers, and policy makers so that it could be useful for planning, making smart decisions, efficient management, and providing sophisticated public vehicle usage.

“Estimated Time of Arrival” (ETA) refers to the amount of time taken by a vehicle to reach its destination. It is a transportation concept that refers to the length of time it takes for any vehicle like bus, ship, helicopter, or emergency service to arrive at its destination [5]. ETA is commonly used to inform travelers about remaining time available before a certain mode of transportation reaches a particular destination. This paper proposes a real-time bus management system that use HCI-based web and mobile application to remind passengers about the estimated bus arrival time at their destination, considering various factors like traffic jams and weather conditions. In addition to ETA, this system also provides a list of all bus stops for a given bus.

Our contribution for this manuscript is listed below.

- We established Intelligent Transportation System to collect, analyze, and identify patterns in the Indian transportation for the benefit of passengers, bus managers, and policy makers.
- We developed Information and Communication technology-enabled bus management system with advanced information processing system.
- We have created modules to track and display the movement of vehicles in real time under the influence of various factors, such as traffic, weather, and time parameters.
- We have recorded the daily trips and the routes including speed and traffic information with movements of vehicles in a series of topographical zones—geofencing.
- We have developed module to deliver information regarding routes, arrival time, trip duration, traffic information, and transit patterns to the passengers.

The rest of the paper is organized as follows. The research papers related to this field are discussed in Section 2. The hardware and the software requirements are discussed in Section 3. The algorithm implemented in the paper is explained in Section 4. The implemented model is compared with other models and necessary illustrations are made in Section 5. The results and the scope for further research are discussed in Section 6.

## 2. Related Work

The Framework Program for Research, Technological Development and Innovation (DESMI 2008) of the Cyprus Research Promotion Foundation focuses more on Event-Based Bus Monitoring System (EBM) [6]. Specifically, the focus is on reducing contact signals in order to produce an acceptable result. Bus arrival times are meticulously tracked. EBM's findings indicate that it hires just 3.5 percent of the overall volume of signaling communications, bringing it down to a manageable level to a major degree.

M-ESB is a multisensor data collection and sharing interface for a handheld sensor grid and router that feeds into a remote data cloud. It also introduces a new network business model in which the public bus company acts as a Virtual Mobile Service provider. The use of WSN technology as a method for managing traffic signals between Johannesburg and Pretoria is defined in Vehicle Traffic Monitoring Using Wireless Sensor Network in South Africa. Furthermore, RFID scanners are used in the system to detect congested areas and warn the traffic officer at the Traffic Monitoring System. The authors presented an integrated system [7] that monitors the current position of the bus/vehicle and indicates the actual position to the regular user, as well as alerting the regular user of any catastrophic event that may cause a natural slowdown during traveling [8]. This information about the bus's approach is stored on a back-end server, and commuters are informed of this information through a mobile application, allowing them to choose an alternative direction [9]. This device infers that the main emphasis is on the position of the buses and the potential delays due to any disaster [10].

Ingle [11] proposed a system aiming to develop a low-cost solution for helping the passengers obtain the information related to their buses and journeys by considering live bus location tracking, showing seating capacity, showing maximum number of standing passengers allowed in the bus, displaying number of passengers currently traveling in the bus, and calculating Estimated Time of Arrival as essential features of the system. The information about the bus fares from one place to another was also provided in the application. The Silver Cloud Real-Time GPS tracker was used in the system for tracking live location of the bus. The data were sent from the GPS tracker to the database through HTTP using POST method. The user interface was provided as a web application developed using JavaScript, MySQL, and Google Map APIs [12].

The designed methodology decreases the time that different users would wait for a bus. The bus can be tracked at any time and from any place using a device. All current data is saved on the server and accessed by remote users via a web-based application. This method allows users to get details directly displayed on a Google Map in a more user-friendly manner [13].

A smartphone application is used to monitor nearby vehicles and to send updates about them [14]. People can schedule their journeys and travel choices based on the proximity of bus stops [15]. It was introduced in order to change people's commuting decisions by taking into account the “Best Transport Division.” [16]. The android application contains necessary details of the vehicle [17].

Luo et al. [18] proposed a framework based on IoT for public transport system integrating the bus, subway, and shared taxi, with their scheduling problems for proving better transfer solutions; additionally, methods are proposed for predicting the transport flow based on periodic patterns mining utilizing the passenger flow analysis and road flow analysis. A decision support system and mathematical model based evolutionary computation algorithm were used for dynamic bus scheduling and controlling problems. This IoT-based system can assist the passengers to utilize the transportation systems effectively and can reduce the travel time.

Chavhan et al. proposed an Internet of Things-based Intelligent PTS (IoT-IPTS) in a metropolitan region [19]. IoT is utilized to interconnect transportation elements, like vehicles, routes (sensors), commuters (cell phones), and side of the road units in a metropolitan territory. The IoT gives consistent connectivity between various networking systems at whatever point the passengers or vehicles move starting from one area to the next area. Subsequently, IoT gives the reasonable and consistent public transportation administrations in the metropolitan territory. Moreover, context data of transportation elements, for example, condition of routes, traffic congestion, number of routes accessible, vehicles movement pattern, and mobility, are stored in the cloud. The stored data in cloud alongside the IoTs are utilized to locate the significant routes, alternative modes, arrival time, departure time, transit planning, and many more for giving public transportation administrations in a metropolitan zone.

Chavhan et al. proposed dynamic vehicle allotment framework for public transportation using Emergent Intelligence (EI) strategy in a metropolitan zone. Also, the EI method's ability for tackling public vehicle framework issues is illustrated. EI method keeps up recorded data, commuters' appearance rates, availability of resources, and deficit in resources; an EI strategy is used to gather, investigate, share, and ideally assign transport resources adequately [20].

Treethidtaphat et al. utilize GPS information from a public transportation line for bus to build up a bus arrival time forecast at any distance along the considered route [21]. Deep learning is utilized to get high accuracy. The accuracy of the model is assessed by real-time BMTA-8 bus transport information in Bangkok, Thailand, and contrasted the outcome with ordinary least square regression model. The result shows that the proposed deep learning model is more precise than the ordinary least square regression model around 55% for mean absolute percentage error. This shows that deep learning could be used as an effective tool for predicting arrival time [22].

Peilan et al. proposed a prediction strategy using bus trajectories, considering bus route and road network [23]. Passengers' multiple trips were accounted for, including waiting time at multiple points. A deep learning-based model is developed for multiway travel time prediction. Various experimental approaches were proposed to validate the supremacy of considered multiway dataset considered. Using an offline bus location data, also some studies were done to predict the bus running time [24]. Based on the prediction, bus schedules were updated.

Even though some of Intelligent Transportation projects are implemented in some cities in India, all these projects are small-scale standalone pilot studies. Even though they are not of integrated nature, significant efforts that have been made for employing ITS in various cities are discussed. It is evident from this scenario that there are several avenues available for ITS application to flourish, in spite of its growing popularity among the transport authorities. Also, it demands a systematic approach. After the ITS application is implemented at road network level, its complete benefits can be seen. It cannot be seen at the small scale or corridor level. On viewing the present transportation context in India, emergency management, congestion management, advanced traffic management systems, advanced traveler information systems, commercial vehicle operations, advanced vehicle control systems, and so on are the aspects that require focus, other than the existing ITS applications.

In all these researches, the systems developed did not use traffic density and weather as factors for influencing the calculation of arrival time. These two factors are the major parameters used as input attributes for the machine learning model to train and predict the Estimated Time of Arrival of the bus in real time.

## 3. Methodology

### 3.1. System Architecture

The major components of the hardware kit comprise of the following:

- Location provider module
- Centralized real-time database
- HCI interface

### 3.2. Location Provider Module

**Figure 1:**
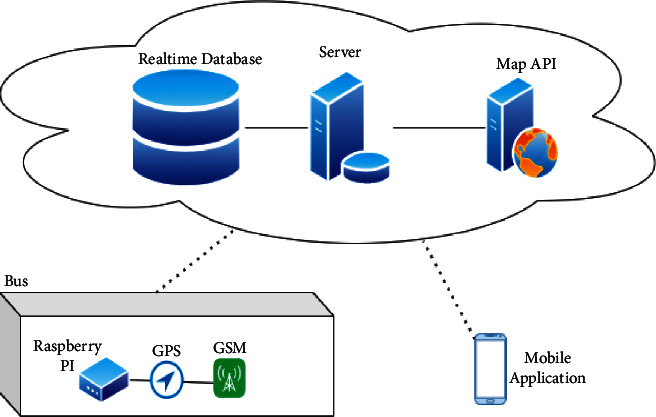
System architecture.

**Figure 2:**
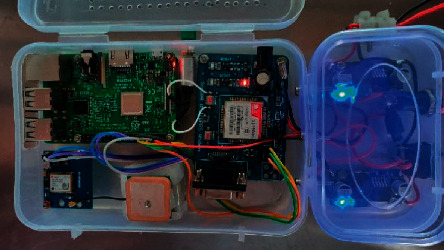
Real-time vehicle tracking module.

The location provider module consists of a GPS module, a GSM/GPRS module, and an IoT controller like Arduino or Raspberry Pi. Here, Raspberry Pi is used for quick development and easier debugging.  Figure 1 presents the overall system architecture and Figure 2 shows the prototype built using the architecture in Figure 1.

### 3.3. GPS-Raspberry Pi Configuration

Once the GPS module is connected to a power source, the module tries to get a position fix with the help of the antenna that searches for any nearby satellites to get the current location of the module. The red led starts to blink once the module gets its location fix. After getting the location fix, the module starts transmitting location and other data in the form of an internationally standardized string that is standardized by NMEA (National Marine Electronics Association). This NMEA formatted GPS data contains number of NMEA messages that represents each type of data transmitted by the satellites. The data is transmitted to the controller module where the NMEA messages get parsed and processed to actual location data.

### 3.4. GSM/GPRS-Raspberry Pi Configuration

Before configuring the GSM/GPRS sim 900 A module with Raspberry Pi, a sim card with sufficient data should be inserted into the sim Module. The sim Module has all the functionalities similar to the features of the mobile phone, like calling, messaging, using Internet, and so on. These functionalities are carried out by AT commands that are passed to the sim Module from the Raspberry Pi controller. These commands instruct the sim Module to turn on data when connection is made with the controller. The ground of the Sim 900 A module is connected to one of the ground pins of Raspberry Pi. The power source of this module is a 12 V DC power supply that can be supplied either with a DC adapter charger to a wall plug or a DC 12 V battery.

### 3.5. Real-Time Database

As the name suggests, a real-time database works as a state machine, triggering certain events when the state of the database, more precisely when a data in the database gets added, updated, or deleted. When the data gets updated, the database synchronizes the data with the clients' local data. In this way, the database sends the updated data to the client instead of client sending a new request every time to the database to acquire the updated data. Google's Firebase Real-Time database is the perfect example of this functionality. The database works under the principle of data synchronization with the clients connected to the database and the data is stored in a nonrelational JSON tree. Each data represents a node in the JSON tree and each node can act as a root node and can have its own tree. This feature of the real-time database is used in a manner where the database sends the response to the connected client whenever the data changes in the database.

### 3.6. HCI Interface

The client interface is a progressive web application that is built using AngularJS. The client shows the current bus stops and the other stops it crossed including the estimated time of arrival of the bus at each stop. The interface consists of three components: a map to show the current bus route and location markers, a side-menu to choose different bus routes, and a sidebar to see the ETA for different stops of the current bus.

The client is connected with the real-time database where the data is synchronized with the client on every update in the database. The map interface is taken from the Google Maps API that provides the location markers and the map of the route of the current bus that is selected. The bus stops are manually created in the database for each bus called as waypoints, where each waypoint denotes a bus stop.

### 3.7. Deployment in Bus

**Figure 3:**
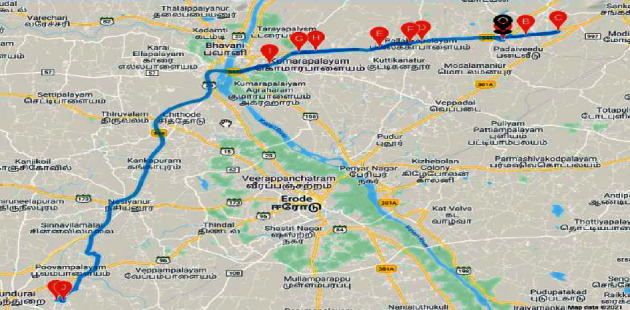
Route 82 roadmap.

The hardware module shown in Figure 2 was deployed in a Kongu Engineering college bus, route number 82. The route 82 has the longest route among all buses running in the college; the source point is the college in Perundurai, and the destination is Sankagiri post office. The route map is shown in Figure 3.

### 3.8. Control Flow

**Figure 4:**
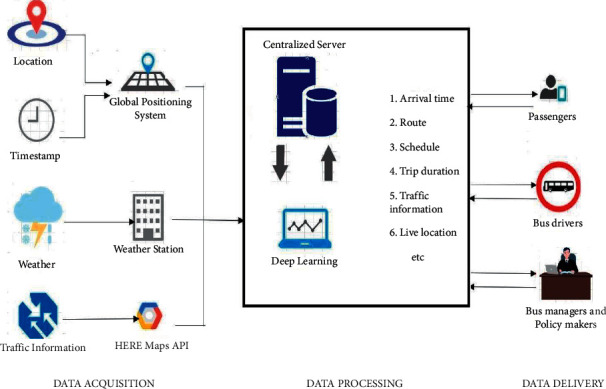
Data flow diagram.

The data and control stream starts with the GPS module and ends with the client interface. The control flow diagram given in Figure 4 helps in determining how the data flows throughout the modules. Once the GPS module gets the location fix, it starts sending the NMEA messages to the Raspberry Pi through the TX pin connected to one of the GPIO pins of Raspberry Pi. The messages are read using PyGPIO python library that reads serial data from the GPIO pins. The controller then parses the NMEA messages to fetch the respective latitude and longitude coordinates of the current location given by the GPS module. The parsed location data is sent as a request to a third-party traffic and weather API that fetches the traffic density and weather data of that location. The fetched traffic data and weather data are then processed by the controller and sent to the real-time database as nonpreprocessed raw data. For the third-party API, HERE Maps API is used to get both weather and traffic data for the location given by the Raspberry Pi controller. Whenever the vehicle crosses one of the waypoints, the controller computes the average traffic density between all crossed waypoints and the distance between the last crossed waypoint and the current way point. The duration between the two waypoints is also taken into account. These data along with the current waypoint location and the crossed waypoint location and names of those waypoints and the time at which the vehicle crossed the last waypoint with the time at which the vehicle arrived to the current waypoint makes up for the ML data that is sent to the real-time database. The accuracy of the location provider module is adjusted for getting the best results in terms of the radius under which the GPS gets the location coordinates and the controller parses the data and the GSM/GPRS module sends the data to the real-time database. The accuracy ranges from 10 meters to 250 meters maximum.

All these data are parsed into JSON objects and serialized to normal strings and sent as each request to the firebase real-time database and to the third-party HERE Maps API. The response contains the weather data of the current location in JSON format. Changing these weather data types into numeric factor can help in training the model.

This weather data is used as one of the factors for training the ML model. Another important factor is the traffic density the HERE Maps API provides. This traffic density varies in the range of 0 to 10 where “0” is the chosen location completely free of traffic and “10” is the chosen location completely full of traffic jams and heavy traffic. This traffic density has a great influence over the ML model that is being trained for the prediction of ETA of the vehicle. After collecting the required data from the HERE Maps API, the JSON responses are appended to the processed ML data and stored in the firebase real-time database. This data acts as input to the ML model for training and testing. The predicted ETA is then stored in the real-time database for the client interface.

The HCI interface fetches the ETA of the current selected vehicle from the list of routes and displays it to the user. Here the change in ETA triggers a change in state of the data in the real-time database which in turn triggers the responses to be sent to all the clients connected with the real-time database. The interface gets updated data every time the real-time database synchronizes the data with the client. As each route is selected from the client interface, the client sends a request to fetch the data from the real-time database. As the vehicle moves, the current location gets updated in the real-time database and triggers data synchronization with the client. The client displays the current location of the vehicle in map with predicted ETA for the next arriving waypoint using the help of Support Vector Regression model.

### 3.9. Support Vector Machine

Support vector machine is a simple classification algorithm that is used to find a hyperplane in an *N*-dimensional space, where *N* is the number of features that distinctly classifies the data points. The input data can be projected to higher dimensions if the data cannot be separated at lower-dimensional space. This can be achieved using Kernel functions. Being able to learn from very small datasets, avoiding local minimums and generalization capability are some advantages of support vector machine [25].

Hyperplanes are the decision boundaries created by the algorithm to classify the data points into different class labels. Margin is the distance between hyperplane and the data points of each class. Kernel function is the function that takes data points as input and transforms them into required form. The different kernel functions that are available are linear, polynomial, radial basis, sigmoid, and so on. It returns the inner product of two data points in a feature space [26].

The support vector machine algorithm finds the equation for the optimal hyperplane from the training data and uses it for later predictions. The confidence value for the classifications will be directly proportional to the distance between the current data point and the hyperplane, which is the decision boundary [27]. The main purpose behind finding this optimal hyperplane that is far from all data points is to maximize the confidence value for future predictions. Consider there are input vectors *x*_*i*_, where *i* ranges from 1 to *n*, reflecting the number of features that affect the result of the algorithm, weight vectors *w*_*i*_ that is the linear combination to classify the class labels or predict the value of *y* in case of regression and *b* for intercept. The value for *y* used by support vector machine is *y* *ϵ* {−1,1} in case of binary labels. For each training example (*x*_*i*_,*y*_*i*_), a corresponding functional margin *Y*_*i*_ of (*w*, *b*) is used to check for the confidence value of each prediction. Here, *w*^*T*^ is the weight vector and *b* is bias term. It can be mathematically seen as given in the following equation:

(1) $$\begin{array}{c} Y_{i}=y_{i}\left(w^{T}x+b\right). \end{array}$$

As already mentioned above, the confidence value will be higher if the margin value is higher. If *y*_*i*_ is 1, then the functional margin value should be large, which in turn depends on (*w*^*T*^*x* + *b*) being larger and positive based on the above equation. In contrast, if *y*_*i*_ is −1, then (*w*^*T*^*x* + *b*) should have larger magnitude but be negative [28]. Therefore, for the most accurate predictions, the functional margin value must be larger than 0 and as close as 1. If it is greater than 0, the prediction might be correct.

(2) $$\begin{array}{c} Y_{i}>0=>y_{i}\left(w^{T}x+b\right)>0. \end{array}$$

To find the decision boundary that maintains maximum distance with each data point of each class, the magnitude should be taken into consideration since it is not possible to calculate the distance without the magnitude. The functional margin is normalized with the Euclidean distance *d*, which is the distance between the data point and the decision boundary. Hence, (1) can be modified as below:

(3) $$\begin{array}{c} Y_{i}=y_{i}\frac{\left(w^{T}x+b\right)}{\left\|w\right\|}. \end{array}$$

By dividing the functional margin by the magnitude, a constraint is posed on the size of *w* that maintains the same value for high values with same proportions, thus normalizing the functional margin. So, the geometric margin that is the Euclidean distance and the distance between the decision boundary and positive boundary can be given as the following equation:

(4) $$\begin{array}{c} \frac{\left(w^{T}x+b\right)}{\left\|w\right\|}=\frac{1}{\left\|w\right\|}\text{ and }\frac{2}{\left\|w\right\|}. \end{array}$$

To maximize 2/||*w*|| is the same as to minimize 1/2*∗*||*w*||*∗*||*w*||, which is a quadratic program that can be solved easier. Hence, the following minimization condition can be given:

(5) $$\begin{array}{c} \text{Minimize}\frac{1}{2}*\left\|w\right\|*\left\|w\right\|\text{such that }y_{i}\left(w^{T}x+b\right)-1\geq 0. \end{array}$$

This problem can be solved using Lagrangian multiplier method since it is constrained quadratic optimization problem by posing a multiplier on the constraint. This multiplier is called as Lagrangian multiplier and this makes the equations at any data point; when the support vector is not present, the value for route function *a*_*i*_ and *a*_*j*_ becomes zero. The equation is given as

(6) $$\begin{array}{c} \text{Min}\left(w,b,\alpha \right)=\frac{1}{2}\left\|w\right\|*\frac{1}{2}*\left\|w\right\|-\sum_{i=1}^{m}a_{i}yi\left(wTx+b\right)\cdots 1. \end{array}$$

Deriving the Lagrangian multiplier from equations (4)–(6) will produce the below equations:

(7) $$\begin{array}{c} w=\sum_{i=1}^{m}a_{i}y,\\ \sum_{i=1}^{m}a_{i}y_{i}=0. \end{array}$$

Maximizing over *a* can produce new Lagrangian equation by getting rid of dependence on *w* and *b* with replacing *w* in the above equations. Thus, the below shown dual optimization problem can be produced.

(8) $$\begin{array}{c} \text{Max}\left(a\right)=\sum_{i=1}^{m}a_{i}-\frac{1}{2}\sum_{i,j=1}^{m}y_{i}y_{j}a_{i}a_{j}\langle x_{i},x_{j}\rangle \text{such that }a_{i}\geq 0,\\ \sum_{i=1}^{m}y_{i}yi=0. \end{array}$$

The above equation shows that the dot product of *x*_*i*_ and *x*_*j*_ influences the maximization of the “*a*” value. When the inner product of *x*_*i*_ and *x* is large and from different classes, it forms the margin with maximum width whereas the inner product of the same class does not yield any significance [29]. The value of *w* and *b* can be in turn obtained by obtaining the *a*-value that maximizes *L*(*a*). Thus, the final equation can be given as

(9) $$\begin{array}{c} w^{T}+b=\left(\sum_{i=1}^{m}a_{i}y_{i}x_{i}\right)Tx+b=\sum_{i=1}^{m}a_{i}y_{i}\langle x_{i},x\rangle +b. \end{array}$$

Since the “*a*” value will be zero for all points except the support vectors, the summation of the classifier equation for all non-support vectors will result in zero. So, the prediction is done by considering only the inner product of support vector and the newly provided *x* which may result in values greater than or equal to zero or less than zero. Greater than or equal to zero means the prediction class is positive and less than zero means the prediction class is negative [30].

Support vector regression works similar to support vector machine and uses the same principles but for regression problems while support vector machines are widely used for classification problems. Support vector regression is not a probabilistic approach and does not assume any randomness. The main objective of the support vector regression algorithm is to find a function that approximates mapping from the input data points to a real number on the basis of the provided training sample. The function defines the hyperplane that can be of any shape and mostly high dimensional based on the number of features affecting the result to produce minimum error. This algorithm also uses concepts used in support vector machine, like the hyperplane, decision boundary, and margin. The same constraints that are used in the support vector machine are used for support vector regression. The constant distance *d* from the hyperplane to the nearest data points defines the equations for decision boundaries. The equations are given as *wx* + *b* = +*d* and *wx* + *b* = −*d* for positive and negative decision boundaries, respectively. Thus, the equation of any hyperplane that satisfies the support vector regression should satisfy the below equation:

(10) $$\begin{array}{c} -d<y-wx+b<+d. \end{array}$$

The algorithm basically considers the data points that are within a decision boundary and produces the best-fit line or plane, which is the hyperplane that has maximum number of points. The margin tolerance *C* and the decision boundary distance *ϵ* can be manipulated by the supervisor for better results with minimum error and producing a better fitting model. From this, support vector regression provides the user the flexibility to define how much error is acceptable in the model and find an appropriate hyperplane to fit the data.

## 4. Developing the Model

The data obtained from the real-time vehicle tracking device is stored on a real-time database. These data are preprocessed and converted into the dataset with the attributes like source location, destination location, distance, average jam factor, average weather, and duration of travel. All these input features are used in training the support vector regression model. The training set contains 75 percentages of the data, and the test set contains the remaining 25 percentage. The transportation data is collected for 30 days. A total of 3360 entries are recorded. Among these, 2520 are used for training the model and 840 are used for testing. Here, the model is trained to provide the duration of travel as a result from the other input features. The model is then integrated with a web server to use the predictions for real purposes.

### 4.1. Data Preprocessing

The data obtained from the real-time vehicle tracking module is the raw data. The data contains only information about the current location, like latitude, longitude, timestamp, jam factor, and weather. The data is then analyzed, and the dataset is generated. Python scripts are written for detecting the data produced at the waypoints of the bus. These data are separated, and another script is written to transform this data into a dataset that contains source, destination, distance, average jam factor. The average jam factor is calculated by taking average of all the jam factors of the data instance generated from source to destination. The average weather is calculated similar to the average jam factor. The duration of travel is calculated by taking difference between the timestamp of the data instance from source and the destination [31].

The dataset is ready now from the theoretical point of view, but to use this dataset with the python functions, the data cannot be strings and all the instances in the dataset should be converted to float values. For this purpose, scikit-learn provides Label Encoder class for converting all the string values into float values. And since all the values present in the dataset may not be at the same range of numbers, they need to be transformed so that all the input features fall in the same range of numbers. For example, the duration is in seconds, so the values can be in thousands range if the bus route is long enough, but the jam factor values range between 0 and 10. It may lead to difficulty in plotting the data points.

This problem is resolved by a technique called feature scaling, where all the input features are transformed to the same range. There are various methods for feature scaling. The min-max scaling is used here. The mathematical formula for min-max scaling is given as

(11) $$\begin{array}{c} x_{i}^{n}=\frac{\left(x_{i}-\min\left(x_{i}\right)\right)}{\left(\max\left(x_{i}\right)-\min\left(x_{i}\right)\right)}. \end{array}$$

**Figure 5:**
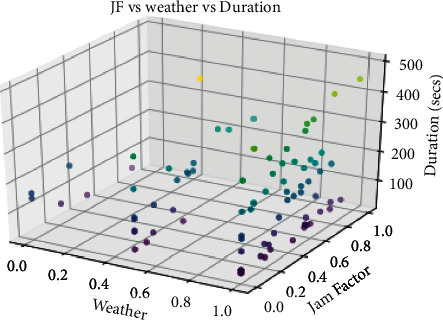
3D Visualization of dataset.

Here, *x*_*i*_^*n*^ is the scaled value, *x*_*i*_ is the *i*th value, and min and max are the minimum and maximum values for the given range. The raw dataset and the processed dataset are shown in Tables 1 and 2. A 3D visualization of the dataset is shown in Figure 5.

### 4.2. Building and Training the Model

The model is developed and trained in the python programming environment using various libraries, like Pandas, Scikit-Learn, NumPy, and Matplotlib. The data from the real-time database is cleansed and converted to a CSV file. The Scikit-Learn module provides the functions for implementing the support vector regression algorithm.

The preprocessed data is separated into two datasets in the ratio of 3 : 1 using test_train_split function provided by the Scikit-learn library. The larger part is fed to the support vector regression model whose implementation method is provided by Scikit-learn library. The model training can be manipulated using four different parameters. The process of changing these parameters is called hyperparameter optimization [32]. The first parameter is *C*, which defines the weight of how much samples inside the margin contribute to the overall error. This allows the user to optimize both the fit of the line to data and penalize the samples inside the margin. It in turn allows the user to adjust how hard or soft the margin classification should be. With high values of *C*, the samples inside the margin are penalized more. The second one is epsilon. Epsilon defines the value of margin where the errors are tolerated and not penalized. The larger the value of epsilon, the larger the number of errors admitted. The third parameter is kernel. Kernel is the function that takes input data and transforms it into the required form of processing data. Kernel function transforms the training dataset so that a nonlinear decision surface is able to be transformed into linear equation in a higher-dimensional space. The return value of this function is the inner product of two data points in a standard feature dimension.

There are different types of kernel functions available like linear kernel that is used when the data points are linearly separable, Gaussian kernel which is used when there is no prior knowledge about the data points, radial basis function which is the most commonly used, sigmoid kernel, polynomial kernel, and so on. The fourth parameter is the gamma. Gamma is the hyperparameter that decides how much curvature the decision boundary can have. The magnitude of gamma is directly proportional to the curvature of the decision parameter.

The values for these parameters used for training the model are chosen by running a python script that trains the model with a range of values for *C* and epsilon. The technique implemented in the script is the grid search technique. The gamma is auto, which considers 1/*n*, where *n* in the number of features and kernel function is radial basis function. These two parameters were constant for all pairs of *c* and epsilon. The model trained with different values of *c* is tested for error. The model with the lowest error is taken for training the final model. Then, epsilon is changed within a range, where *c* is the resultant of previous step and the model with lowest error is found. This value of epsilon is used for final model. The final model is trained using the *C* and epsilon values that resulted in the previous process and the other parameters remain as default.

## 5. Testing the Model

The trained model is tested for accuracy with one-quarter of the dataset. Unlike classification problems, regressions do not produce absolute binary values but rather they provide a numeric value in a range as a result. There are various metrics to measure the errors produced by an algorithm.

### 5.1. Mean Squared Error (MSE)

This method measures the average of squares of the error, which means average of the differences between the actual value and the predicted value. The result will always be nonnegative. The results closer to zero are better.

(12) $$\begin{array}{c} \text{MSE}=\frac{\sum_{i=1}^{n}{\left({\text{AT}}_{i}-{\text{PT}}_{i}\right)}^{2}}{n}. \end{array}$$

Here, AT_*i*_ is the actual time, PT_*i*_ is the predicted time, and *n* is the number of predictions.

### 5.2. Mean Absolute Error (MAE)

This is the simplest error measurement method where the error is calculated as the average of the absolute differences between the actual values and predicted values. It is mathematically given as

(13) $$\begin{array}{c} \text{MAE}=\frac{\sum_{i=1}^{n}\left({\text{AT}}_{i}-{\text{PT}}_{i}\right)}{n}. \end{array}$$

Here, AT_*i*_ is the actual time, PT_*i*_ is the predicted time, and *n* is number of predictions.

### 5.3. Root Mean Square Error (RMSE)

This is a quadratic-based rule to measure the absolute average magnitude of the error. It is calculated by summing all the differences between actual values and the predicted values, squaring the difference value and dividing the sum with the number of predictions and finally taking a square root of the value. Since the values are squared and rooted, the result will always be positive. The mathematical formula is given as

(14) $$\begin{array}{c} \text{RMSE}=\sqrt{\frac{\sum_{i}^{n}{\left({\text{AT}}_{i}-{\text{PT}}_{i}\right)}^{2}}{n}}, \end{array}$$

where AT_*i*_ is the actual time, PT_*i*_ is the predicted time, and *n* is number of predictions.

### 5.4. Relative Absolute Error (RAE)

This method is similar to the mean absolute error, but instead of using actual value, a simple predictor is used to provide with values in place of actual values.

### 5.5. Relative Squared Error (RSE)

This method compares the model with a simple predictor. Total squared error of the tested model is normalized and divided by total squared error of the simple predictor.

This trained prediction model is tested for accuracy using Mean Squared Error and Root Mean Squared method. And grid sliding technique is employed to find the least error possible hyperparameters.

## 6. Comparison with Other Models

**Figure 6:**
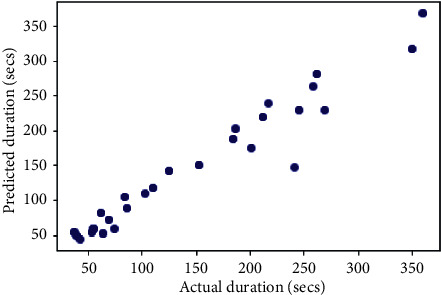
Predicted versus actual plot for SVR.

**Figure 7:**
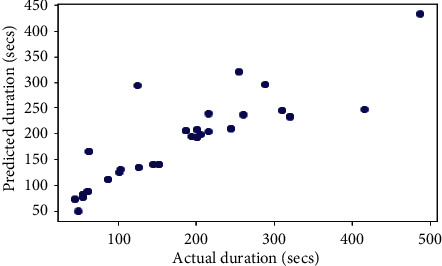
Predicted versus actual plot for RFR.

**Figure 8:**
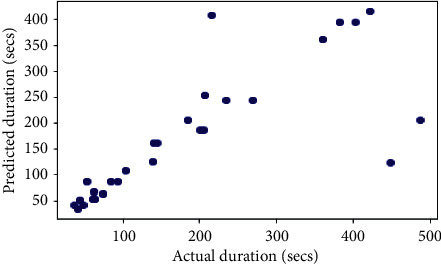
Predicted versus actual plot for DTR.

**Figure 9:**
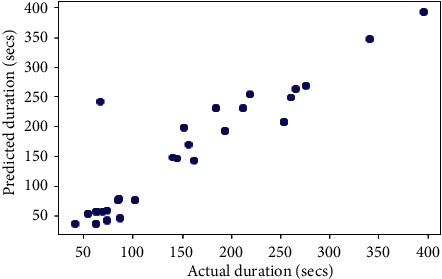
Predicted versus actual plot for KNNR.

**Figure 10:**
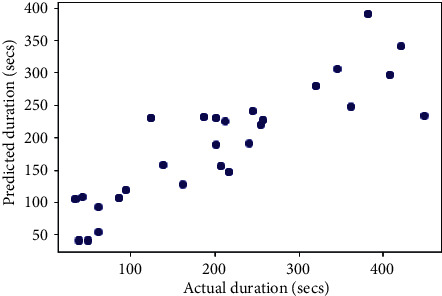
Predicted versus actual plot for GBR.

**Figure 11:**
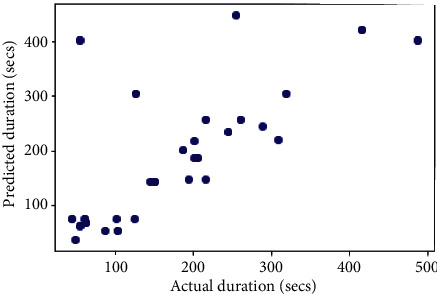
Predicted versus actual plot for XGBR.

**Figure 12:**
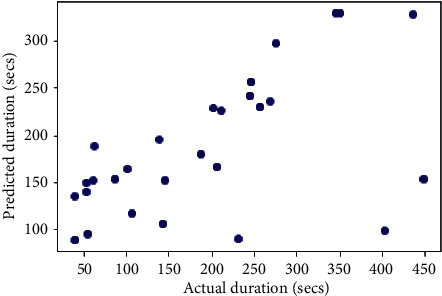
Predicted versus actual plot for AdaBR.

The same dataset was used for training and testing different models so that we can compare the performance of SVR with others. The algorithms chosen for other models were Random Forest Regressor, Decision Tree Regressor, *K*-Neighbors Regressor, Gradient Boosting Regressor, XGB Regressor, and AdaBoost Regressor. The results produced by the models are shown in Table 3. Graphs of actual duration against predicted duration were plotted for each of the models. The linearity in the graph shows the accuracy of prediction. So, if the graph is as linear as the line *y* = *x*, then the model works with 100 percent accuracy. The actual by predicted plot is a scatter plot. The predicted response (*Y*-hat) is used for the abscissa. The observed response (*Y*) is used for the ordinate. The plot can also be used to visually evaluate the possibility of “lack of fit.” An unbiased prediction should produce predicted values that agree with the observed values on average. If the model is biased, then the data points will deviate from the line. For example, if the response to changing the factors is nonlinear but the model includes only terms for linear effects, then the model will be biased. Figures 6–12 show the predicted versus actual plot for SVR, RFR, DTR, KNNR, GBR, XGBR, and AdaBR, respectively.

**Figure 13:**
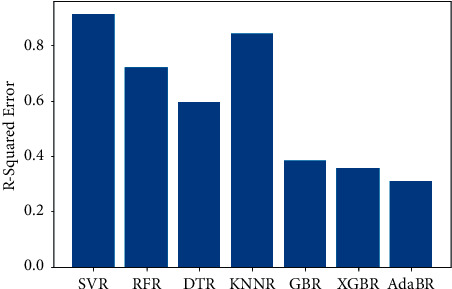
Comparison of *R*-squared error.

**Figure 14:**
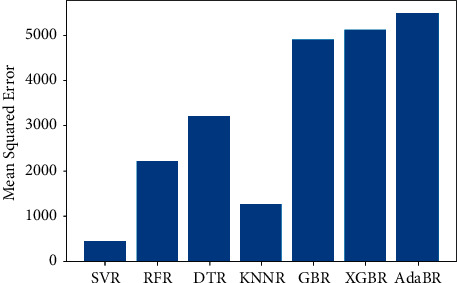
Comparison of mean squared errors.

**Figure 15:**
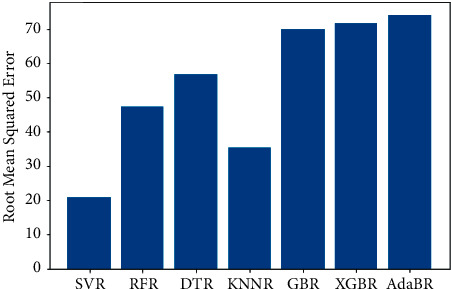
Comparison of root mean squared errors.

Figures 13–15 represent the comparison of *R*-squared, mean squared error, and root mean squared error for regression algorithms considered. All the data shown in the table are visually represented in the bar graphs. The *y*-axis in those bar graphs represents error value by the respective error measuring metric and the *x*-axis represents the different models measured for error. From all the scattered point graphs of predicted values against actual values, the model with support vector regression algorithm produces results quite resembling the linear graph whereas in other models the points were a bit more scattered. From Table 3, it is clear that support vector performs the best among all the taken models with an accuracy of 92 percentages. *K*-Neighbors Regression was kind performing nearly as good as Support Vector Regression with only 15 seconds of more RMSE than SVR. But when it comes to real-time usage, the model might have to predict the ETA continuously while the buses are being operated. So, choosing the KNNR algorithm will result in a lot of computation and prediction will be very slow since it is a lazy algorithm and storing all training data would require more memory. Random Forest regression was the better performing model next to KNNR with an RMSE of 47 seconds. This RMSE is more than twice of that of SVR. The usage of Random Forest regression for this case would have been better if the data set consisted all possible values for every feature. Since the dataset is being generated in real time, a data instance with certain weather and a certain jam factor may not even be obtained before the situation occurs. That means a jam factor of value 10 will occur when the road is fully jammed and filled with vehicles where nothing can move. This situation never occurred while gathering the data. So, when using RFR, the model may not be able to extrapolate that may lead to very poor predictions. The other models did not perform well and the RMSE were very much higher compared to SVR.

## 7. Result Analysis and Conclusion

After testing the model with one-fourth of the total dataset, the model was producing results with Root Mean Square Error of 20 seconds. The accuracy of the predictions is better when the RMSE value is as low as zero. The error shown by the model is less than 0.5 minutes and that is very low compared to the duration of the travel time of the bus. The travel time of the bus deployed with the remote bus tracking module is nearly one and half hours, which is 90 minutes. So, comparing the error with the duration, the error is very much small. It is less than 0.1 percent of the duration. But when compared to the duration between waypoints, it will be 6–8 percent since the average duration between waypoints is 6–7 minutes. This result was achieved by tuning the hyperparameters of the Support Vector Regression algorithm using the grid search technique.

This model considers the jam factor and weather conditions, which are not considered in other models. From similar researches, it can be observed that other models have 40–50 seconds of error for each station. Comparatively, this model performed better with only 20 seconds RMSE. The Support Vector Regression algorithm implemented here predicted Estimated Time of Arrival (ETA) accurately and the real-time bus tracking module facilitated gathering the information effectively.

There is still some room for improvements. The first one would be using high configuration components for the real-time bus tracking module like better GPS module and GSM that supports 4G data communication. The second improvement would be reducing the load of the Raspberry Pi by making all the API calls from separate server so that Raspberry Pi only focuses on transmitting the location data. Next improvement would be using bigger dataset for training the model to achieve better performance from the model. This improvement can be made easily but requires quite a lot of time to generate all the real-time data.

Both mobile- and web-based application will be developed to predict the trip duration of the buses. These applications will be hosted in the website so that bus travelers, bus drivers, and bus owners can use the app and accurately predict the location of the buses. It is also proposed to give an alarm if the bus is struck with an accident or traffic jam. Any user with less proficiency can also use the system as it is a simple easily accessible system. The dataset collected will be published online for future research purpose.

The major challenge in real-time implementation will be the networking between all the modules, the database, and users. The next challenge will be performance tuning, when using the same model for predicting in all routes. This paper can be implemented as a real-time application with some changes like performance tuning in the model, training the model with more data, and providing users with additional features in the website and a dedicated mobile application. This can also be extended for other transportation systems. It can be very useful in industries for supply chain management.

## Figures and Tables

**Table 1:** Raw dataset.

Latitude	Longitude	Date	Time	Jam factor	Weather
11.45428433	77.8137363	11-03-2021	07 : 21 : 06	1.02058	Passing clouds
11.45513267	77.81313	11-03-2021	07 : 33 : 26	1.80532	Passing clouds
11.27909933	77.59135	11-03-2021	08 : 19 : 52	0.02995	Fog
11.27546733	77.58735	11-03-2021	16 : 58 : 09	2.50171	Partly sunny
11.45908733	77.839009	11-03-2021	17 : 59 : 38	3.92654	Scattered clouds

**Table 2:** Processed dataset.

Source	Destination	Average jam factor	Average weather	Duration (seconds)
Goundanoor	ICL PO	2.33693	Scattered clouds	309
Goundanoor	KPR school	3.07268	Scattered clouds	245
Katheri road	Kottaimedu bus stop	1.389322	Passing clouds	186
Katheri road	Kottaimedu bus stop	4.66105333	Scattered clouds	254
KPR school	Muniyappan Kovil	0	Scattered clouds	345
KPR school	Muniyappan Kovil	0.017798333	Scattered clouds	350

**Table 3:** Comparison of different models.

Algorithm	*R*-Squared	MSE	RMSE
SVR	0.914788192823	433.41898834	20.8187172
RFR	0.721238437404	2221.3996912	47.13172701
DTR	0.597991021123	3203.5357142	56.59978546
KNNR	0.842112238521	1258.17857142	35.47081295
GBR	0.38344528449	4913.21128311	70.09430278

## Data Availability

The data used to support the findings of this study are included within the article.
